# Applying the WHO’s framework for age-friendly environments: older adults’ perspective in a rural setting

**DOI:** 10.1093/geront/gnag015

**Published:** 2026-02-15

**Authors:** Anna Nivestam, Ellinor Edfors, Gita Hedin, Maria Haak, Albert Westergren

**Affiliations:** Faculty of Health Sciences, Department of Nursing and Health Sciences, Kristianstad University, Kristianstad, Sweden; Faculty of Health Sciences, Department of Nursing and Health Sciences, Kristianstad University, Kristianstad, Sweden; Faculty of Health Sciences, Department of Public Health, Kristianstad University, Kristianstad, Sweden; Faculty of Health Sciences, Department of Nursing and Health Sciences, Kristianstad University, Kristianstad, Sweden; Faculty of Health Sciences, Department of Nursing and Health Sciences, Kristianstad University, Kristianstad, Sweden; Department for Research, Development and Education, Management for Psychiatry and Habilitation, Region Skåne, Lund, Sweden

**Keywords:** Digitalization, Discrimination, Healthy aging, Rural environment, Transport

## Abstract

**Background and Objectives:**

The World Health Organization (WHO) has outlined eight domains central to age-friendly environments. Since their introduction in 2007, developments have focused mainly on urban areas. This study explores which factors older adults view as crucial for creating age-friendly environments in a rural Swedish context.

**Research Design and Methods:**

This qualitative study consisted of five focus groups with 17 participants. A deductive approach was used for the analysis.

**Results:**

Discussions were linked to all eight WHO domains, with a particular emphasis on digitalization, transportation, and discrimination, especially related to digital and social inclusion, which cut across several domains. For instance, digitalization influenced both transportation and communication in rural areas with long distances and limited services. Social inclusion and non-discrimination highlighted the need for greater adaptation within specific domains, especially for people with mobility limitations, declining health, or financial difficulties. Overlaps among domains were evident, such as the need for accessible and affordable transport to enable healthcare access and social participation.

**Discussion and Implications:**

To create age-friendly rural environments, adaptations are needed within all domains in the WHO framework. Digitalization should be emphasized to enable inclusion and reduce discrimination. Accessible and affordable transportation is also crucial for older adults to participate socially, engage civically, and access healthcare. The WHO framework appears sufficient in a rural context. Therefore, we suggest using the framework for age-friendly environments in practice to consider aspects of the environment that older adults perceive as important.

## Background and objectives

Age-friendly environments are crucial for promoting healthy aging. The World Health Organization (WHO) defines healthy aging as “the process of developing and maintaining the functional ability that enables well-being in older age” (WHO, 2015, p. 28). Healthy aging is influenced by a person’s intrinsic capacity (e.g., cognitive function, mobility, and vision) and surrounding environments. These environments can range from the home and buildings to societal attitudes toward older adults and political decisions (WHO, 2015). To highlight the impact of environments on health and well-being, the WHO outlined eight domains for an age-friendly city in their 2007 guide (WHO, 2007). This guide was later adapted to a European context in 2017 (WHO, 2017), changing the term to “age-friendly environments,” which encompasses both urban and rural areas. This shift is particularly relevant for rural settings, where limited access to services and infrastructure can pose unique challenges for healthy aging.

In Europe, the proportion of older adults aged 60 years or more living in rural areas varies widely, from 15% to 60% depending on the region (WHO, 2024). In Sweden, the corresponding figure is 15% (WHO, 2024), and most municipalities are sparsely populated, with more than half of residents living outside urban clusters of over 50,000 inhabitants (Statistikmyndigheten [The Statistical Authority], 2015).

The eight domains for age-friendly environments are (WHO, 2017): (1) *Outdoor environments* focus on the accessibility and usability of public spaces and buildings for older adults. It emphasizes the importance of barrier-free environments, availability, and safety. Adaptations should facilitate social interaction and physical activity. (2) *Transport and mobility* aim to improve infrastructure for walking and cycling, accessible public transport, and tailored transportation services. (3) *Housing* focuses on home adaptations, support to age in place, and alternative housing. (4) *Social participation* encourages access to a variety of social activities and lifelong learning. (5) *Social inclusion and non-discrimination* emphasize activities to combat ageism and promote intergenerational learning. (6) *Civic engagement and employment* focus on older adults’ contributions through meaningful activities and engagement in society. (7) *Communication and information* addresses access to clear and understandable information, health literacy, and the digital gap. (8) *Community and health services* focus on integrated care, health promotion, and support for informal caregivers. It also emphasizes services that are accessible and tailored to the needs of older adults. To some degree, the content of the different domains overlaps (WHO, 2017).

Previous research highlighted that these domains are sufficient to use for working on the creation of age-friendly environments (Steels, 2015). However, in the latest updated version (WHO, 2017), older adults were not asked to discuss the age-friendly domains and their content (as was done in the 2007 guide). Instead, the focus was on how local authorities have reacted to what was outlined in the 2007 WHO global guide (WHO, 2017). In addition, much of the existing research on age-friendly environments has focused on urban contexts (Buffel & Phillipson, 2016; Van Hoof et al., 2018), while rural environments remain comparatively underexplored (Montayre et al., 2022). Seminal work by Keating (2008) underscored the distinct challenges of rural aging, such as limited transportation options and sparse access to services, but over 15 years have passed since that analysis, and only a few recent studies have revisited these issues in rural settings, mostly in the United States, Canada, and Australia (Colibaba et al., 2020; Liebzeit et al., 2023). During this time, substantial societal shifts have occurred. In particular, the rapid rise of digital technology has transformed everyday life, often introducing new barriers for older adults (United Nations, 2019). For example, digital fraud disproportionately affects older people (Kemp & Erades Pérez, 2023), and unequal access to technology continues to exclude vulnerable subgroups (Gaber et al., 2025; Suntai & Beltran, 2023). These developments illustrate how digitalization intersects with other forms of inequality, such as gender, race/ethnicity, and socioeconomic status, shaping older adults’ ability to age well (Pickard et al., 2025; Yeh, 2022). Therefore, it is important to critically examine how age-friendly frameworks function in rural areas, where both structural constraints and intersectional vulnerabilities may be especially pronounced.

Given the broader and more inclusive understanding of age-friendly environments, it is important to examine how the WHO’s framework applies in rural contexts. Including the perspectives of older adults is essential to fully assess the framework’s relevance. This study therefore aims to explore what older adults themselves view as essential for creating age-friendly environments in a rural Swedish context, guided by the WHO’s framework for age-friendly environments.

## Research design and methods

### Design

Qualitative data were collected and analyzed, employing focus group discussions as the primary method of data collection, guided by Krueger’s methodological framework for focus group research (Krueger, 1988; Krueger & Casey, 2015). This study used a descriptive qualitative design to explore how the WHO’s framework for age-friendly environments aligns with the lived experiences of older adults in a rural southern Swedish context. While the interview guide was informed by the WHO framework, participants were not introduced to its terminology. Instead, open-ended questions allowed participants to share experiences freely. The subsequent analysis examined how these reflections corresponded with, and provided contextual detail to, the WHO domains, with a focus on identifying particularities of the Swedish rural setting. This approach emphasizes the co-construction of knowledge through participant interaction, where individuals build on each other’s reasoning to generate new insights (Dahlin Ivanoff & Hultberg, 2006; Krueger & Casey, 2015).

### The setting

The study was conducted in five rural municipalities in southern Sweden, each participating in a regional collaboration on preventive home visits for older adults. These municipalities have populations ranging from approximately 10,000 to 50,000, with about 30% of residents aged 60 years or older (Statistikdatabasen [The Statistical Database], 2024). The municipalities are, in general, characterized by long travel distances to services and limited public transport options, features typical of Swedish rural areas.

### Sample and procedure

Purposive sampling (Patton, 2015) was used to select participants through a research and collaboration project named “Preventive Home Visits to Seniors” (Pre-H). This municipal service, offered independently of the present study, targets older adults—typically around 77 years of age, but also including those younger or older—aiming to assess needs and promote health and well-being before more serious issues arise (Nivestam, 2022). Eligible participants were community-dwelling older adults aged 65 years or more, living in non-institutionalized private residences such as single-family homes, villas/detached houses, or apartments, without municipal home help or care services.

Gatekeepers—five individuals in key community or institutional roles, one from each of the participating municipalities—provided study information during preventive home visits in spring 2024. Interested individuals received written information, confirmed their willingness to be contacted, and had their names and phone numbers forwarded to the research team. The second author then telephoned potential participants to give further information and schedule focus groups. Participation was entirely voluntary and had no connection to access to preventive home visit services.

A total of 27 participants were initially recruited for the study. Of these, 10 later declined—three during scheduling, three due to illness or a funeral, two due to other commitments, and two without providing a reason. In the end, 17 participants took part in the study, each attending one of five focus group discussions, one held in each of the five participating municipalities. Group sizes ranged from two to six participants. Table 1 presents the demographics of the study sample. The median age is reported, as this better reflects the central tendency than the mean in a relatively small sample.

**Table 1:** Demographics of the study sample.

Characteristics	Sample (*n* = 17)
**Gender, *n***	
Women	10
Men	7
**Age, years, median (range)**	77 (76–81)
**Highest education, *n***	
Secondary education	10
Bachelor’s degree	4
Master’s degree	3
**Previous work experience, *n***	
Health and social care	5
Information technology	1
Teaching	2
Construction	4
Transport	1
Military	1
Government agency	3

### Data collection

Five focus group discussions, conducted from April to June 2024, were held close to the participants’ locations, grouping them by municipality. The first author, who was experienced in moderating focus groups, led the discussions using a guide covering the main themes: Important aspects for healthy aging and the surrounding environment and important aspects to communicate to societal actors to promote healthy aging. While the interview guide was informed by the WHO framework for age-friendly environments, it was not structured strictly according to its eight domains. Instead, open-ended questions were used to allow participants to discuss their experiences freely in relation to healthy aging and the surrounding environment.

The moderator went through the informed consent form to ensure that the participants understood it and had signed it. The participants were asked to fill in background information (age, gender, previous work, and education). The moderator and co-moderator (the second author) briefly introduced themselves and their roles, emphasizing that the participants were the experts, and their perspectives would drive the discussions. Time was given for participants to ask questions before starting.

The audio recording began after consent was received from all participants. The participants briefly introduced themselves by name and shared their experiences with the preventive home visit. The moderator then introduced the first question from the discussion guide, followed by the others. Follow-up questions were asked, such as: Can you explain more? What do you others think about that? After each session, a debriefing between the moderator and co-moderator was conducted to reflect on important discussions. The discussions lasted between 1.5 and 2 hr each and were transcribed verbatim using Amberscript (n.d.), a transcription software. While the software was used to produce initial transcriptions, each transcript was carefully reviewed and verified by members of the research team to ensure accuracy. This included checking for potential errors caused by unclear speech, accents, or overlapping dialogue. Any discrepancies were corrected by comparing the transcript with the original audio recordings.

Elements such as reflective note-taking and member checking were also used to enhance the trustworthiness of the findings. These notes captured contextual details, group dynamics, and emerging impressions and were used to complement the transcripts by clarifying ambiguous statements and identifying potential themes.

### Data analysis

Krueger’s approach to focus group analysis (Krueger, 1988; Krueger & Casey, 2015) was used, emphasizing the identification of shared meanings produced through group interaction rather than individual accounts. The first and second authors reviewed the transcripts and audio recordings multiple times to gain a holistic understanding of the discussions. This iterative reading focused on recurring ideas, areas of agreement and divergence, and how participants built on one another’s contributions, central features of Krueger’s analytic method (Krueger, 1988). During this process, reflective notes taken during and after each focus group supported the interpretation by highlighting dynamics and clarifying ambiguities.

As the transcripts were repeatedly examined, it became evident that participants’ reflections naturally aligned with the domains in the WHO framework for age-friendly environments (WHO, 2017). Although the framework was not originally intended as the analytical lens, its relevance emerged inductively during the early stages of analysis. Consequently, the research team adopted a deductive orientation in the organization of data, using the WHO framework to structure categories. This deductive step was guided by methodological literature on deductive analysis (Graneheim et al., 2017; Patton, 2015), but the analytical procedure itself followed Krueger’s approach (Krueger, 1988; Krueger & Casey, 2015). In other words, Krueger’s method guided *how* shared meanings were identified, while the WHO framework guided *where* in the analytical structure these meanings were placed.

NVivo 2020 software was utilized to organize the material. During coding, the first and second authors identified illustrative examples discussed within the focus groups, for instance, “Traveling for free on public transport avoided issues with apps” and “With reduced mobility, it is difficult to get to shops and public transport.” These examples were then categorized according to the eight domains of the WHO framework. The second author critically reviewed the coding, and any discrepancies were discussed until agreement was reached.

Next, the first and second authors synthesized descriptive meanings from each domain, ensuring that interpretations remained grounded in group-level interactions rather than individual statements. The third, fourth, and fifth authors contributed by listening to the focus group discussions and offering their interpretations, which were integrated into the emerging analysis. A preliminary summary of the findings was developed by the first author and reviewed by the full research team before being shared with participants via email or mail.

A member-checking process was conducted in November 2024 with 14 participants (three declined), following the principles of trustworthiness described by Lincoln and Guba (1985). Participants received a written summary of the preliminary thematic interpretations and were invited to provide feedback. Three participants joined an online seminar to discuss the findings, and the second author conducted follow-ups with the remaining 11 participants. All participants confirmed that the interpretations resonated with their experiences, and several suggestions, such as further elaboration on the meaning of security in outdoor environments, were incorporated into the final analysis. The final results were agreed upon by all authors.

The COREQ checklist (Tong et al., 2007) was followed to guide transparent and comprehensive reporting of the study design and procedures.

### Ethical considerations

All participants provided written informed consent prior to participating in the study. In addition, verbal consent was obtained at the beginning of each focus group session to ensure that participants had fully understood the study information and their rights, including the option to withdraw at any time. This step served as an ethical safeguard and an opportunity to reconfirm their willingness to participate. Ethical approval was obtained from the Swedish ethical review authority (reference number 2022-06054-01).

## Results

An initial reading of the transcripts revealed that three issues—digitalization, transportation, and discrimination—were particularly prominent in participants’ reflections in the rural context studied. This was confirmed in the deductive analysis, which organized all discussions within the eight WHO domains for age-friendly environments and showed these themes cutting across multiple areas. Digitalization was linked to both transportation (e.g., app-based ticketing) and communication, while experiences of social inclusion and non-discrimination were shaped by factors such as limited mobility, declining health, and financial hardship. Participants noted that those with stable health and finances faced fewer barriers and that several domains overlapped, e.g., transportation was essential for both healthcare access and social participation in rural areas.

### Outdoor environments

Across all groups, safe and accessible outdoor spaces were seen as essential for supporting both physical activity and social interaction. In these rural municipalities, proximity to nature was often an advantage for those who were mobile, yet safety concerns—such as encountering unfamiliar people or being far from help if ill or injured—could limit use. Perceptions of safety varied; in some areas, police initiatives were said to have improved security. Participants also valued opportunities for socializing in outdoor community settings, such as affordable cafés or meeting places, though they noted that such options had become scarce:

We have such wonderful nature around here. Yes, it’s really great. Mhm. And getting there… well, you can take the bus to the nature center in […]. There are beautiful trails there, some easy to walk and others more challenging. And here in the village, you can walk along the river. (FG2)

It’s terrible [the feeling of safety when moving about]. You often read in the XX newspaper about 80- and 75-year-olds being tricked… But I must say, one is more observant now than a few years ago. I often walk in the forest for exercise, and if I meet some guys, then and there I become a bit more cautious […] when someone looks a bit suspicious. (FG5)

Exercise facilities were appreciated when spacious, accessible, and tailored to older adults, suggesting that well-designed physical environments can facilitate both activity and connection.

### Transport and mobility

Participants stressed that reliable, accessible, and flexible transport mattered more than cost. While some municipalities offered free public transport for older adults, limited routes, infrequent schedules, and distance to bus stops restricted its usefulness—especially for those with reduced mobility or living in remote areas. Digital-only ticketing and parking systems were a major barrier:

It’s not the cost. It’s the service. […] Since they started with apps and such, many people can’t handle that aspect anymore. […] Standing there with parking. They can’t solve the ticket because it has changed [a new app or update]. (FG4)

Desired improvements included more frequent and better-routed buses, alternative payment methods (cash or card), and continued availability of flexible options such as subsidized taxi services. Many relied on private cars but feared isolation once driving was no longer possible. Strict eligibility for paratransit was also seen as excluding those in need.

I think it’s important to highlight accessibility [to transportation]. […] They just make it more and more difficult, and then it becomes so troublesome and complicated. So no, I stay at home instead—and that’s not good. (FG4)

### Housing

Across the groups, housing discussions centered on the need for age-adapted homes—both sheltered housing (independent living units for older adults that include some shared services or staff presence but not full-time care) and nursing homes—located near services, with good accessibility, reasonable costs, and available support for aging in place. Participants wanted more sheltered housing that combined private apartments with shared spaces and optional communal lunch services. They also valued being active contributors to housing life, such as cooking together or helping plan activities.

We live in a split-level house […] we couldn’t live more cheaply, since anything else would be about twice the rent. But of course, one would like a ground-floor apartment with a little green space outside—that would be ideal, along with being close to town. (FG5)

Concerns were raised about the criteria for nursing home admission, with calls for age and loneliness to be considered alongside physical health. A shortage of short-term care homes (temporary residential facilities providing care following hospital discharge or during periods of increased need, often used as a bridge to returning home or transitioning to long-term care) sometimes forced people to live in other municipalities, complicating family visits. The perceived closure of specialized dementia care homes was seen as leading to more mixed care arrangements, which caused insecurity about appropriate care.

I’m talking about the old lady […] she was 97. She applied for a care home after a fall and feeling unsafe at home, but was rejected for not being ‘ill enough.’ I mean, at 97, why can’t you choose? […] Why can’t they be allowed to have a good life in the years they have left? (FG1)

To remain in their own homes, participants wanted practical help with tasks such as gardening and cleaning. Many homes were not mobility-adapted, raising concerns about future modifications. While some had already adapted their homes or moved to more suitable residences, others noted a shortage of ground-level apartments (single-floor residences with direct outdoor access, often preferred in the Swedish context for their accessibility and suitability for aging in place) and secure housing. They urged municipalities to build more such apartments, ideally with small gardens and located near essential services, but stressed that prices must remain affordable, as new rental and owner-occupied units were often too costly for pensioners.

Yes, those new ones [ground level apartments for older adults] are not cheap, you know. (FG1)

### Social participation

Participants emphasized that social activities must be affordable, accessible, and inclusive, spanning physical, social, and cultural opportunities. While some municipalities offered a good range of activities, others were seen as lacking, and there was concern that those most in need—such as people experiencing loneliness—were not always reached.

Meeting places are important. […] When a spouse passes away, the one left behind needs this much more than when living as a couple. It’s important to have somewhere to go for a meal, or for things like book talks. And it shouldn’t just be for those who are alone—it should be available for everyone. (FG2)

Not all older adults sought organized activities; some preferred family contact or solitary pursuits. Positive examples included municipalities where older adults had “ownership” of activities, helping decide, arrange, or lead them while the municipality provided the venue and administrative support. As one participant described a popular local gathering:

It’s cheap. You go there and have a coffee and a sandwich, it’s really cheap […]. They [other older people] sit there for an hour or so and they have their own table in some corner and discuss and […] solve the seriousness of life. (FG5)

### Social inclusion and non-discrimination

Participants identified multiple factors that can exclude older adults—such as loneliness, bereavement, functional decline, limited finances, and rural isolation—and stressed the need for environments adapted to these challenges. Direct outreach to lonely individuals was seen as ineffective if framed solely around loneliness; instead, participants suggested approaches that begin with practical help, like grocery shopping, to build trust before addressing social needs. Those who had lost a partner were viewed as particularly vulnerable, and ideas such as neighborhood help—where youths or older adults assist with errands—were proposed, offering potential for intergenerational exchange. As one participant explained:

They have tried different things, but it doesn’t work in this municipality. […] It becomes too exposed [not private] […] You have to feel it. Because it has to do with personal chemistry. […] Some kind of neighborhood help […] You know your area. […] You know roughly who lives there. (FG4)

Exclusion was also linked to impairments like hearing loss or reduced mobility, as well as structural barriers including limited rural public transport, digital-only services, and the high cost of participation or technology for those on low pensions. These combined personal and structural barriers were seen as reinforcing inequality and limiting participation in community life.

### Civic engagement and employment

Participants valued a vibrant association life for fostering participation and social cohesion but noted challenges in sustaining it. Recruiting volunteers—particularly from the “younger older” generation—was difficult due to competing priorities, limited time, and a shift toward expecting payment. As one participant reflected:

You can say, I’ve been involved in association life ever since I was little. […] It has shrunk. I mean, it takes a lot of volunteer efforts. […] To get people to work voluntarily or…? People want to get paid. […] But we have been very involved in both the dog club and the riding club. (FG1)

Some maintained lifelong interests through associations, while others stepped back from leadership roles with age. Courses and training, often led by older adults themselves, were seen as mentally stimulating.

Nowadays I only work as a study circle leader, with cooking classes in the municipality. […] Last Saturday we had a course called “Making your own sausage.” It’s always nice and appreciated. (FG1)

Beyond associations, participants wanted their willingness to contribute to society recognized and actively sought out—whether through managing a home or garden, caring for livestock, reading to nursing home residents, or looking after grandchildren. Such activities were seen as meaningful for both the individual and the community.

Sometimes you feel it’s so good for your health to do a good deed… just helping someone with something… you feel good from being able to do something for someone else. (FG4)

### Communication and information

Digitalization shaped much of the discussion, with participants describing both opportunities and significant barriers. While some in the “new older generation” were familiar with technology through work, others faced physical, cognitive, or financial obstacles to using smartphones, bank ID, or online services—leading many to rely on family for digital tasks. Exclusion arose when digital was the only option, especially for accessing healthcare, banking, or public services. As one participant noted:

She went to the bank and spent a morning there talking to everyone. […] She went to the health center a lot and talked to them. But she felt that they don’t have time for that. […] Home care is also like that. Just in and out as quickly as possible. (FG1)

If you live alone and only have a small pension, you can’t afford to go and buy a mobile phone for several thousand kronor [several hundred dollars]. (FG4)

Human contact was preferred over chatbots or online platforms, with traditional mail and especially direct phone calls seen as the best ways to communicate—particularly about health matters. Participants lamented the loss of everyday social interactions that once took place in places like bank offices and expressed growing concerns about fraud in digital transactions, suggesting safeguards such as longer transfer times. These experiences underscored a need for secure, user-friendly systems and for retaining analog communication options to prevent exclusion.

### Community and health services

Participants emphasized continuity, accessibility, affordability (understood as both rising care costs and the financial strain on those with limited pensions), and equal access to assistive devices. Seeing the same healthcare providers was a priority—whether a “family doctor” at the clinic or familiar staff in home care—since inconsistency caused anxiety:

It is important that home care works. And maybe that can be brought forward. That not too many different people come to you. […] When different people come every time who do not know you. (FG2)

Geriatric care clinics, where nurses could be reached easily by phone, were appreciated.

Assistive devices such as hearing aids, walkers, and mobility scooters were seen as essential, but their cost varied greatly across municipalities; participants wanted more subsidies and national price uniformity. Rising home care costs and perceived inequalities deterred some from using services. Other concerns included the reduction of safety teams that support patients after hospital discharge, fear of developing dementia and a wish for regular cognitive testing, and the declining quality of municipal food deliveries, which discouraged continued use.

…it’s about special transport, costs and everything. It should be the same across the whole country. It shouldn’t differ […] whether you live in XX or the neighboring municipality. The same with whether you get a free hearing aid or not. (FG4)

## Discussion and implications

While the focus group discussions reflected key areas of the WHO framework for age-friendly environments (WHO, 2017), they also underscored specific challenges particularly relevant in rural contexts, most notably digitalization, transportation, and discrimination. Importantly, participants were not presented with the framework terminology during discussions, yet their experiences naturally aligned with several of its domains. This supports the framework’s general relevance while suggesting a need for contextual adjustments to better address the realities of rural life, such as incorporating analog communication alternatives, designing transportation systems that function without smartphone-based apps, and actively addressing digital literacy and access. The observed interdependence among domains, such as transportation enabling social participation and healthcare access, further highlights the importance of integrated strategies tailored to rural conditions.

Accessible transport is central to age-friendly environments and remains a pressing concern in rural contexts. Older adults in the present study described a noticeable decline in public transport services, which directly impacted their ability to access healthcare, engage socially, and maintain independence. These concerns echo findings in the literature about rural transport disadvantages and aging populations (Ravensbergen et al., 2022; Zhang & Yang, 2024). Additionally, the study sheds light on how digitalization further complicates access. App-based ticketing systems were viewed as exclusionary by many participants, particularly those unfamiliar with or unable to use digital technology due to physical, cognitive, or financial barriers. While digitalization has the potential to support inclusion and access to services, our findings highlight that for many older adults in rural contexts it often acts as a barrier, leading to exclusion from essential services such as healthcare, public transport, and banking when digital tools are the only option. These concerns are echoed in recent studies emphasizing the persistent digital divide among older adults in rural settings (Fischl et al., 2020; Lee et al., 2021). These intersecting challenges suggest that transport policy and infrastructure must address both physical and digital accessibility to truly support age-friendliness in rural areas.

In addition to access barriers, the affordability of digital devices and services emerged as a recurring concern, intersecting with broader issues of social inequality. Previous research indicates that digitalization can lead to both positive and negative experiences for older adults (Hill et al., 2015). Some studies highlight positive effects such as increased inclusion and social participation (Fischl et al., 2020; Sen et al., 2022), particularly when digital tools are tailored to older adults’ needs and supported by adequate training and social contexts. However, when digitalization is implemented without such support, it may reinforce existing disparities and result in exclusion. The cumulative effect is a form of digital discrimination, where those least able to adapt are most likely to be excluded. Addressing this requires not only technical solutions but also a stronger policy focus on equity and rural digital inclusion.

Age-friendly environments should consider specific groups in society, such as people experiencing loneliness, financial difficulties, and disabilities, or those living in rural areas. The older adults in this study emphasized that support must be sensitive to the complexity of these challenges, particularly in rural contexts where services may be less visible or accessible. Research shows that it can be difficult to talk about loneliness or express that one feels lonely (Neves & Petersen, 2025). Participants suggested that indirect contact, such as assistance with daily tasks, could be more effective in building trust than direct interventions framed around loneliness. One way to reach people who experience loneliness is through preventive home visits. These visits allow for a broader conversation about health and well-being, where loneliness may emerge once trust has been established. This is supported by studies indicating that older adults find these visits trustworthy and relationship-focused (Blotenberg et al., 2023; Nivestam et al., 2025). Moreover, information gathered during these visits can inform municipal planning to promote healthy aging (Nivestam et al., 2021, 2026). For example, if loneliness or transport difficulties are commonly identified among older adults in rural areas, decision-makers could respond with tailored interventions. In this way, preventive home visits can serve not only as individualized support but also as tools for addressing structural and contextual challenges in rural environments.

While Sweden’s welfare state provides relatively generous supports—such as subsidized healthcare, preventive home visits, and in some municipalities, free public transport for older adults—our findings reveal that gaps persist in rural contexts. A possible explanation for why climate did not emerge as a concern among participants is the relatively high standard of living in Sweden, together with reliable heating systems, municipal snow clearance, and legal requirements for property owners to keep pavements clear of snow and ice. These conditions likely reduce the perceived impact of cold temperatures in southern Sweden. However, even with such support, free bus travel offers little benefit when routes are infrequent or inaccessible to those with mobility limitations, and preventive home visits may not address the structural barriers created by digital-only services. This contrast underscores that even in well-resourced welfare systems, rural-specific adaptations are necessary to achieve the goals of age-friendly environments.

### Methodological considerations

A strength of this study is that the WHO framework was not introduced to participants during the focus groups, yet their discussions naturally aligned with the framework’s domains. This suggests that the framework resonates with older adults’ lived experiences in rural settings. However, we recognize that our deductive approach, grounded in the WHO framework, may have limited the emergence of themes outside this structure.

The sample included participants from five different municipalities, which added geographic diversity, especially given Sweden’s self-governed municipal system (Kommunallag [The Swedish Local Government Act], SFS, 2017). However, the relatively small number of participants and the focus on healthy older adults without municipal home care services may have restricted the scope of the findings. Including participants with a broader range of health statuses or care needs could have provided additional insights.

To enhance credibility, we conducted a member-checking process in which participants reviewed preliminary analytical themes and provided feedback that contributed to the refinement of our interpretations.

Future research should further examine how individual characteristics, such as ethnicity, social class, and gender, influence engagement with age-friendly environments. Although sampling aimed for variation in gender, education, and work experience, the analysis did not explore how these characteristics shaped perceptions. These social locations intersect and may create specific barriers to participation and digital access (Gaber et al., 2025; Kimpel et al., 2024; Suntai & Beltran, 2023). Gendered experiences, particularly related to transportation, also warrant further study and may require different designs, such as gender-specific focus groups. Applying an intersectional lens is crucial for identifying and addressing structural barriers and ensuring more equitable interventions (Pickard et al., 2025; Yeh, 2022).

## Conclusion

To enable healthy aging in a rural Swedish setting, it is crucial to address specific challenges that older adults face, particularly digital and age-related discrimination and limited transportation options. These issues emerged as central concerns across multiple domains of the WHO framework and highlight the need for rural-sensitive adaptations. Digital services must be complemented by analog alternatives to ensure inclusion, especially for those with limited access or ability to use technology. Discrimination, whether related to age, digital literacy, or rural marginalization, must be actively countered in service design and community planning. Transportation systems should be made both physically and digitally accessible to enable mobility and social participation. While the WHO framework (WHO, 2017) offers a useful structure, this study suggests that rural implementation requires a sharper focus on these intersecting challenges. We therefore recommend using the framework as a guiding tool, while also prioritizing local tailoring that reflects the lived realities of older adults in rural environments.

## Data Availability

The datasets generated and analyzed during the current study were not preregistered and are not publicly available due to confidentiality but are available from the corresponding author on reasonable request.
